# Mutation of the N-Terminal Region of Chikungunya Virus Capsid Protein: Implications for Vaccine Design

**DOI:** 10.1128/mBio.01970-16

**Published:** 2017-02-21

**Authors:** Adam Taylor, Xiang Liu, Ali Zaid, Lucas Y. H. Goh, Jody Hobson-Peters, Roy A. Hall, Andres Merits, Suresh Mahalingam

**Affiliations:** a Institute for Glycomics, Griffith University, Gold Coast Campus, Queensland, Australia; b Australian Infectious Diseases Research Centre, School of Chemistry and Molecular Biosciences, The University of Queensland, Queensland, Australia; c Institute of Technology, University of Tartu, Tartu, Estonia; Johns Hopkins Bloomberg School of Public Health

## Abstract

Mosquito-transmitted chikungunya virus (CHIKV) is an arthritogenic alphavirus of the *Togaviridae* family responsible for frequent outbreaks of arthritic disease in humans. Capsid protein, a structural protein encoded by the CHIKV RNA genome, is able to translocate to the host cell nucleolus. In encephalitic alphaviruses, nuclear translocation induces host cell transcriptional shutoff; however, the role of capsid protein nucleolar localization in arthritogenic alphaviruses remains unclear. Using recombinant enhanced green fluorescent protein (EGFP)-tagged expression constructs and CHIKV infectious clones, we describe a nucleolar localization sequence (NoLS) in the N-terminal region of capsid protein, previously uncharacterized in CHIKV. Mutation of the NoLS by site-directed mutagenesis reduced efficiency of nuclear import of CHIKV capsid protein. In the virus, mutation of the capsid protein NoLS (CHIKV-NoLS) attenuated replication in mammalian and mosquito cells, producing a small-plaque phenotype. Attenuation of CHIKV-NoLS is likely due to disruption of the viral replication cycle downstream of viral RNA synthesis. In mice, CHIKV-NoLS infection caused no disease signs compared to wild-type CHIKV (CHIKV-WT)-infected mice; lack of disease signs correlated with significantly reduced viremia and decreased expression of proinflammatory factors. Mice immunized with CHIKV-NoLS, challenged with CHIKV-WT at 30 days postimmunization, develop no disease signs and no detectable viremia. Serum from CHIKV-NoLS-immunized mice is able to efficiently neutralize CHIKV infection *in vitro*. Additionally, CHIKV-NoLS-immunized mice challenged with the related alphavirus Ross River virus showed reduced early and peak viremia postchallenge, indicating a cross-protective effect. The high degree of CHIKV-NoLS attenuation may improve CHIKV antiviral and rational vaccine design.

## IMPORTANCE

CHIKV is a mosquito-borne pathogen capable of causing explosive epidemics of incapacitating joint pain affecting millions of people. After a series of major outbreaks over the last 10 years, CHIKV and its mosquito vectors have been able to expand their range extensively, now making CHIKV a human pathogen of global importance. With no licensed vaccine or antiviral therapy for the treatment of CHIKV disease, there is a growing need to understand the molecular determinants of viral pathogenesis. These studies identify a previously uncharacterized nucleolar localization sequence (NoLS) in CHIKV capsid protein, begin a functional analysis of site-directed mutants of the capsid protein NoLS, and examine the effect of the NoLS mutation on CHIKV pathogenesis *in vivo* and its potential to influence CHIKV vaccine design. A better understanding of the pathobiology of CHIKV disease will aid the development of effective therapeutic strategies.

## INTRODUCTION

Chikungunya virus (CHIKV) is an arthritogenic alphavirus of the *Togaviridae* family transmitted to humans by mosquitoes of the *Aedes* genus. Urban transmission (human-mosquito-human) can spread CHIKV rapidly within human populations, causing outbreaks of chikungunya fever (CF). CF is a disease characterized by fever, a maculopapular rash, myalgia, and debilitating polyarthralgia, similar in severity to that seen in patients with rheumatoid arthritis (1). CF is usually self-limiting and nonfatal; however, fatalities and severe forms of CHIKV disease have been reported in recent outbreaks, with complications during infection often found in patients with comorbidities. In addition, studies suggest that a high proportion of patients infected with CHIKV experience chronic rheumatic symptoms or relapses of CF that can occur years after the initial infection (2, 3). Following a series of outbreaks that began in Kenya in 2004, the regions of circulating CHIKV quickly expanded from long-established areas of endemicity in East/Central Africa, Asia, and West Africa, now making CHIKV a virus of global concern (4). In 2013, CHIKV spread to the Americas, where local transmission has been identified in over 40 countries or territories. This outbreak has resulted in more than 1 million suspected cases of CF.

CHIKV has a positive-sense single-stranded RNA (ssRNA) genome that contains two open reading frames (ORFs): the nonstructural ORF (nsP1, nsP2, nsP3, and nsP4) and the structural ORF (capsid, E3, E2, 6K/TF, and E1). Both ORFs are translated as polyproteins, which undergo *cis* and *trans* cleavage to form the mature viral proteins. The CHIKV infectious particle comprises a nucleocapsid core containing genomic RNA surrounded by a host-derived lipid bilayer embedded with the E2-E1 glycoproteins. The RNA genome associates with 240 copies of the capsid protein to form the icosahedral nucleocapsid (5). The capsid protein, encoded by the 5′ region of the structural ORF, is capable of autoproteolytic cleavage from the structural polyprotein as the carboxyl-terminal part of capsid protein contains a serine protease (6). The N-terminal region of capsid protein is required for multimerization of the protein to form the nucleocapsid and contains a highly positively charged RNA-binding domain vital for encapsidation of the genome (7–9). Signal motifs in the N terminus have also been shown to be important in the nuclear/cytoplasmic translocation of capsid protein and nucleolar targeting (10–12). In encephalitic alphaviruses, such as Venezuelan and eastern equine encephalitis viruses, the multifunctional capsid protein is responsible for host transcription inhibition and subsequent cytopathic effect (13, 14). Nuclear trafficking of capsid protein was shown to be required for this transcriptional shutoff effect (15). In the arthritogenic alphaviruses examined, host transcriptional shutoff and translational shutoff are thought to be dependent on nsP2 rather than capsid protein (16, 17).

Indeed, despite an exclusively cytoplasmic replication cycle, both encephalitic and arthritogenic alphavirus capsid proteins have been found to traffic to the host cell nucleus. Studies indicate that nuclear trafficking of capsid protein in encephalitic alphaviruses impacts virulence and pathogenesis *in vivo* (15, 18). In contrast, little is known of the importance of capsid protein nuclear, or indeed subnuclear, trafficking in arthritogenic alphaviruses such as CHIKV. Importantly the functional significance of capsid protein subcellular trafficking during CHIKV replication and pathogenesis has not been investigated. Here, using site-directed mutagenesis, we identify amino acid residues in the N-terminal part of CHIKV capsid protein required for nucleolar localization in mammalian cells and perform a functional analysis of mutant capsid protein. Furthermore, we examine the growth kinetics and pathogenicity of a CHIKV containing mutations in the nucleolar localization sequence (NoLS) of capsid protein.

## RESULTS

### Localization of CHIKV capsid protein to the nucleolus and identification of the NoLS.

**FIG 1:**
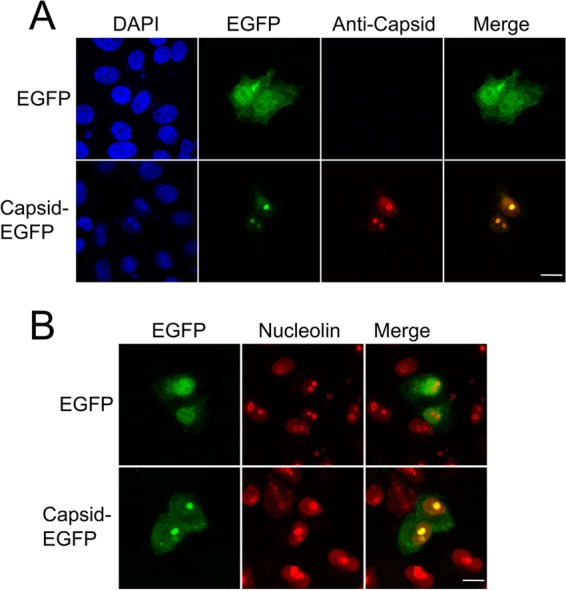
CHIKV capsid protein localizes to the nucleolus. Vero cells were transfected with pEGFP or pCapsid-EGFP. At 24 h posttransfection, cells were fixed and permeabilized, EGFP fluorescence was analyzed by direct visualization, and indirect immunofluorescence was performed using capsid protein-specific antibodies (A) and nucleolin-specific antibodies (B) to identify the nucleolus. Images are representative of at least 6 fields of view. DAPI, 4′,6-diamidino-2-phenylindole. The size bars represent 15 µm.

Previous studies have shown a substantial amount of alphavirus capsid protein translocates to nucleolus-like structures in host cells (10, 11). To investigate the subcellular localization of CHIKV capsid protein, Vero cells were transfected with enhanced green fluorescent protein (EGFP) or EGFP-tagged wild-type (WT) capsid protein expression plasmids. To ensure capsid protein remained fused to the N terminus of EGFP, the C-terminal tryptophan residue (W261) of capsid protein required for capsid protein autoproteolytic cleavage was removed (19); this manipulation had no effect on capsid protein subcellular localization (data not shown). After 24 h, cells were fixed and permeabilized, and capsid protein localization was visualized directly via EGFP fluorescence. EGFP-tagged capsid protein subcellular localization was confirmed with capsid protein-specific antibodies and indirect immunofluorescence (Fig. 1A). As seen previously, in addition to being present in both the cytoplasm and the nucleus, capsid protein showed a prominent localization to nucleolus-like structures. To confirm that these subnuclear structures were indeed nucleoli, indirect immunofluorescence was used to identify the nucleolus using nucleolin-specific antibodies. Nucleolin is a nucleolar phosphoprotein that influences synthesis and maturation of ribosomes and is widely used as a nucleolar marker. Colocalization with the nucleolar marker nucleolin confirmed that CHIKV capsid protein is indeed localizing to the nucleolus (Fig. 1B).

**FIG 2:**
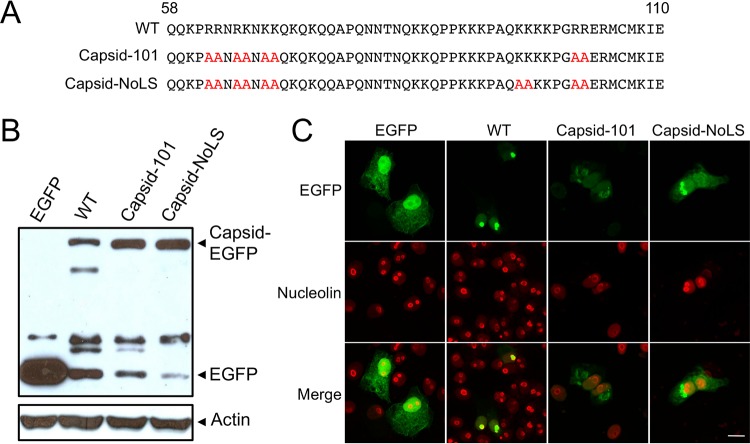
Identification of the nucleolar localization sequence of CHIKV capsid protein using site-directed mutagenesis. (A) WT amino acid residues that make up the putative NoLS were mutated to alanines (highlighted in red). Vero cells were transfected with pEGFP, pCapsid-EGFP, pCapsid-101-EGFP, or pCapsid-NoLS-EGFP. At 24 h posttransfection, cells were either (B) lysed and cell lysates analyzed by Western blotting using EGFP and actin-specific antibodies or (C) fixed and permeabilized, with EGFP fluorescence analyzed by direct visualization and indirect immunofluorescence performed using nucleolin-specific antibodies. Images are representative of at least 6 fields of view. The size bars represent 15 µm.

Bioinformatics, deletion analysis of CHIKV capsid protein (data not shown), and previous studies of alphavirus capsid protein subcellular trafficking have identified a region in the N-terminal part of CHIKV capsid protein, between residues 58 and 110, rich in basic amino acids as the region containing the putative nucleolar localization sequence (NoLS) (11). The bipartite NoLS of Semliki Forest virus capsid protein, between amino acid residues 66 to 83 and 92 to 105, shares basic amino acid sequence homology to CHIKV capsid protein (11). To identify the NoLS of CHIKV capsid protein, site-directed mutagenesis was performed, mutating selected basic residues of WT protein to alanines (Fig. 2A). To determine that mutant forms of capsid protein were expressed at similar levels to WT capsid protein, transfected Vero cell lysates were assayed by Western blotting using an EGFP-specific antibody. The results show that the mutants Capsid-101 and Capsid-NoLS were expressed at least at the same level as WT capsid protein (Fig. 2B).

Upon transfection of Vero cells with plasmids expressing EGFP-tagged WT or mutant capsid proteins, it was found that capsid mutant Capsid-101, although still localizing to the nucleolus, accumulates in the nucleolus at much reduced levels compared to WT capsid protein (Fig. 2C). The fluorescence intensity of Capsid-101 in the nucleolus was comparable to that seen in the rest of the nucleus. Capsid-NoLS was absent from the nucleolus, lacking colocalization with the nucleolar marker nucleolin, and remained largely in the nucleus and cytoplasm. The NoLS of CHIKV capsid protein therefore constitutes 10 basic amino acids located in the N-terminal region of the protein.

### FLIP indicates mutations in the NoLS of capsid protein also inhibit its nuclear trafficking ability.

**FIG 3:**
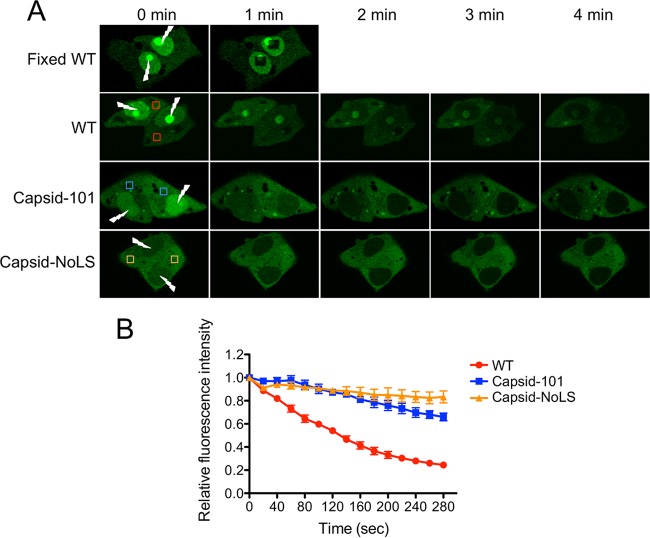
Mutation of capsid protein NoLS inhibits capsid protein nuclear trafficking. FLIP analysis was performed on Vero cells transfected with either pCapsid-EGFP, pCapsid-101-EGFP, or pCapsid-NoLS-EGFP. (A) Fluorescence loss at the region of interest (ROI [colored squares]) in the cytoplasm was measured over a 4-min period during continual photobleaching of the nucleus (lightning bolt). (B) Quantification of fluorescence loss at the cytoplasmic region of interest during a 280-s period of continual photobleaching of the nucleus. To construct fluorescence loss curves, the quantification of the relative fluorescence intensity was carried out using LSM 510 software; the initial fluorescence intensity was set as 1. Each data point represents the mean ± standard error from at least 3 independent replicates.

Previous studies have shown that capsid protein is actively transported to and from the nucleus (10). Having identified the NoLS of capsid protein, we sought to investigate whether mutation of the NoLS affected capsid protein nuclear import trafficking. WT and mutant capsid protein trafficking from the cytoplasm to the nucleus was investigated using fluorescence loss in photobleaching (FLIP) to continuously photobleach a defined portion of the nucleus in live Vero cells expressing EGFP-tagged capsid protein at 24 h posttransfection. Fluorescence loss at the cytoplasmic region of interest (ROI) was normalized using the relative fluorescence intensity from the LSM 510 software, and the initial fluorescence intensity was set as 1. Continuous photobleaching of the nucleus of WT capsid protein-expressing cells resulted in the loss of fluorescent signal in the nucleus and substantial loss of fluorescence at the ROI in the cytoplasm, indicating that cytoplasmic to nuclear trafficking occurred (Fig. 3A). Mutant Capsid-101 and Capsid-NoLS proteins, however, displayed a distinct inability to traffic to the nucleus, as evidenced by the marginal decrease in the relative fluorescence intensity of the cytoplasmic ROI (Fig. 3B). After 280 s of photobleaching, the ROI of Capsid-NoLS shows the smallest decrease in relative fluorescence intensity, suggesting Capsid-NoLS nuclear trafficking is dramatically inhibited. These results suggest that, in addition to inhibiting nucleolar localization, mutation of the NoLS of capsid protein also inhibits capsid protein nuclear import trafficking.

### Mutation of the NoLS did not affect capsid protein autoproteolytic cleavage.

**FIG 4:**
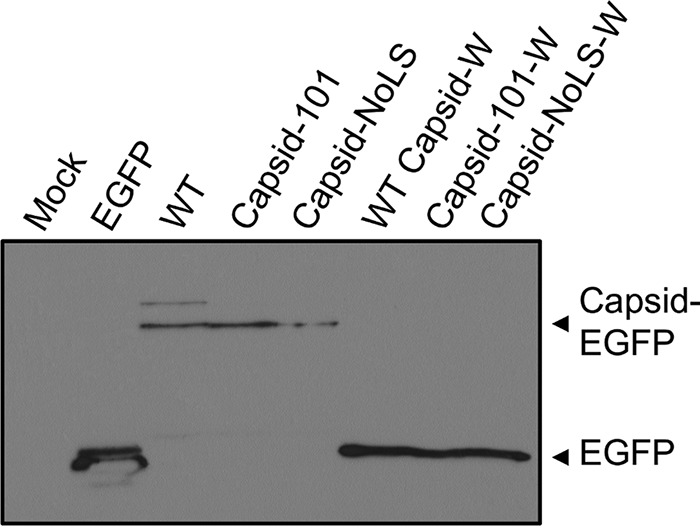
Mutation of capsid protein NoLS does not affect capsid protein autoprotease activity. Vero cells were transfected with pEGFP, pCapsid-EGFP, pCapsid-101-EGFP, or pCapsid-NoLS-EGFP or plasmids expressing EGFP-tagged WT Capsid-W, Capsid-101W, and Capsid-NoLS-W. EGFP-tagged WT Capsid-W, Capsid-101W, and Capsid-NoLS-W contain the C-terminal tryptophan (W261) required for capsid protein autoproteolytic cleavage. Cells were lysed at 24 h posttransfection, and cell lysates were analyzed for cleavage of EGFP from capsid protein by Western blotting and EGFP-specific antibody.

We next analyzed if mutation of the NoLS retained capsid protein autoprotease activity. Constructs expressing EGFP-tagged WT Capsid-W, Capsid-101W, and Capsid-NoLS-W, containing the C-terminal tryptophan (W261) required for capsid protein autoproteolytic cleavage, were generated. Figure 4 shows that all constructs lacking W261 residue from the conserved cleavage site were unable to cleave EGFP from capsid protein. However, capsid protease efficiently cleaved EGFP from capsid protein in all constructs that contained W261, including the NoLS mutants of capsid protein (Fig. 4). Thus, mutation of the NoLS has no effect on the autocatalytic protease activity of CHIKV capsid protein.

### CHIKV containing the NoLS mutation in capsid protein shows attenuation *in vitro*.

**FIG 5:**
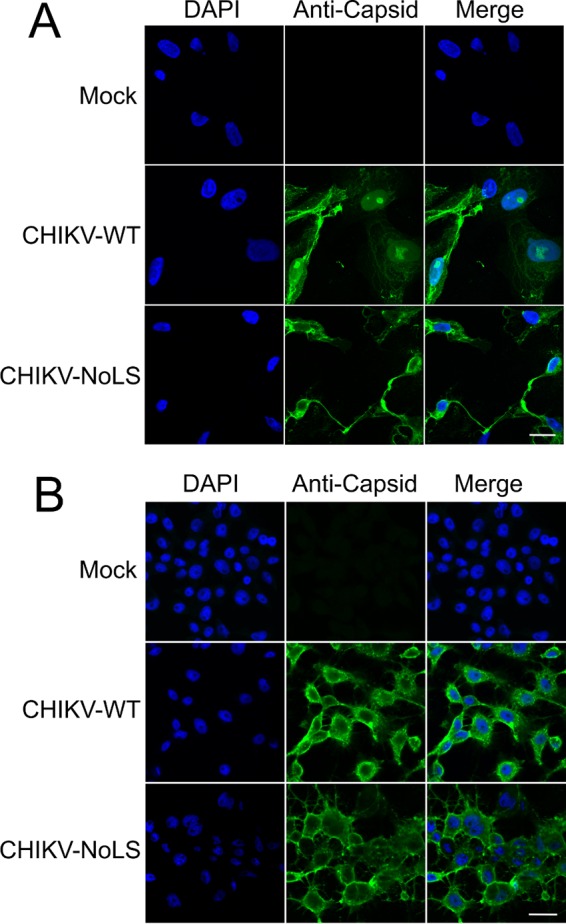
Subcellular localization of capsid protein in CHIKV-WT- or CHIKV-NoLS-infected mammalian and mosquito cells. Vero (A) or C6/36 (B) cells were infected with CHIKV-WT or CHIKV-NoLS at an MOI of 1 PFU/cell. Cells were fixed and permeabilized at 24 h postinfection, and indirect immunofluorescence was performed using capsid protein-specific antibodies. Images are representative of at least 6 fields of view. The size bars represent 15 µm.

To assess the importance of capsid protein nucleolar localization on CHIKV replication, we examined the effect of the NoLS mutation in the context of a full-length CHIKV infectious clone-derived virus. CHIKV containing the NoLS mutation in capsid protein (CHIKV-NoLS) was rescued and propagated in Vero cells. Plaque purification of virus and Sanger sequencing of the entire CHIKV genome confirmed the NoLS mutation was maintained in passaged virus in the absence of additional mutations. Indirect immunofluorescence, using capsid-specific antibodies, was used to analyze the subcellular localization of CHIKV capsid protein in CHIKV-WT- and CHIKV-NoLS-infected Vero cells and mosquito (*Aedes albopictus*)-derived C6/36 cells. Results show that in CHIKV-WT-infected Vero cells, capsid protein accumulates in subnuclear bodies reminiscent of the nucleolus at 24 h postinfection (Fig. 5A). In CHIKV-NoLS-infected Vero cells, these puncta are absent. Thus, in the context of the virus the NoLS mutation causes similar disruption of capsid protein subnuclear localization in infected Vero cells. The NoLS mutation is therefore stable in the virus, resulting in a phenotypic disruption of capsid protein subnuclear localization. Interestingly, in CHIKV-WT-infected C6/36 cells, capsid protein did not accumulate in subnuclear bodies and was found predominantly in the cytoplasm at 24 h postinfection (Fig. 5B). In CHIKV-NoLS-infected C6/36 cells, capsid protein was also found to predominate in the cytoplasm, similar to the localization observed in CHIKV-WT-infected C6/36 cells. Subnuclear localization of capsid protein is therefore not a characteristic of CHIKV infection in insect cells, and mutation of the NoLS has no effect on the subcellular localization of capsid protein in infected C6/36 cells.

**FIG 6:**
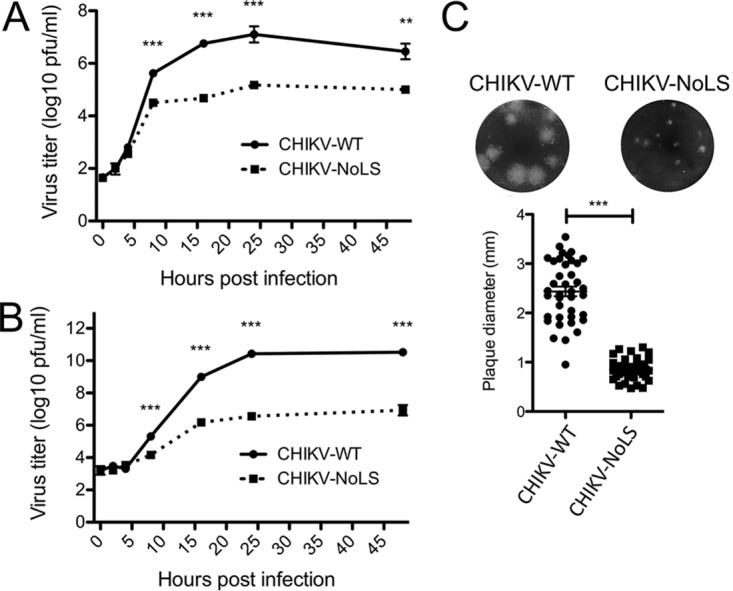
CHIKV containing the NoLS mutation in capsid protein shows attenuation *in vitro*. Multistep growth kinetics in BHK-21 (A) and C6/36 (B) cells were obtained by infecting cells with CHIKV-WT or CHIKV-NoLS at an MOI of 0.1 PFU/cell. Supernatants were collected at the indicated time points, and infectious virus was quantified by plaque assay. **, *P* < 0.01, and ***, *P* < 0.001, by two-way analysis of variance (ANOVA) with Bonferroni posttests. Each symbol represents the mean ± standard error from 3 independent experiments. (C) Plaque size (millimeters) in infected BHK-21 cells. ***, *P* < 0.001, by Student’s unpaired *t* tests. Each symbol represents the diameter of a single plaque.

To examine the replication kinetics of CHIKV-WT and CHIKV-NoLS in mammalian (BHK-21) and mosquito (C6/36) cells, cells were infected at a multiplicity of infection (MOI) of 0.1 PFU/cell, and multistep growth kinetics were analyzed. CHIKV-NoLS grew to significantly lower titers than CHIKV-WT in both BHK-21 cells (Fig. 6A) and C6/36 cells (Fig. 6B). Furthermore, CHIKV-NoLS had a small-plaque phenotype in BHK-21 cells (Fig. 6C), indicating a reduced ability of the virus to spread from the initial site of infection and thus attenuation. To further investigate the attenuation of CHIKV-NoLS in mammalian and insect cells, complementary reverse transcription-quantitative PCR (RT-qPCR) analysis of virus genome copy number in the culture supernatants and virus RNA copy number in infected cells was performed. Results suggest that synthesis of viral RNA remains unperturbed by the NoLS mutation in both BHK-21 (see Fig. S1A in the supplemental material) and C6/36 (Fig. S1B) cells. Furthermore, by 12 h postinfection, the copy number of CHIKV-NoLS RNAs in infected BHK-21 (Fig. S1A) and C6/36 (Fig. S1B) cells significantly exceeded those in CHIKV-WT-infected cells. The difference is potentially due to increased survival of CHIKV-NoLS-infected cells allowing prolonged or more efficient synthesis of viral RNA and/or due to reduced competition between viral replicase and capsid protein for binding viral genomic RNAs. However, the genome copy numbers of CHIKV-NoLS in culture supernatants did not show any increase up to 12 h postinfection in both BHK-21 (Fig. S1C) and C6/36 (Fig. S1D) cells, indicating that no or very little virus was released. This result correlates with the delayed release and reduced titers of infectious CHIKV-NoLS recovered from culture supernatants (Fig. 6A and B). In contrast, for CHIKV-WT-infected cells, virus titers started to increase at 8 h postinfection. The results suggest that although synthesis of viral RNA in infected cells was not reduced, mutation of the capsid protein NoLS causes a defect in infectious virus particle formation, causing reduction and delay of release of viral progeny. Together, these data suggest that subnuclear localization of CHIKV capsid protein is not a hallmark of infection across different host cells and that the attenuation of CHIKV-NoLS in both mammalian and insect cells is likely the result of a defect in infectious virus particle formation due to the NoLS mutation.

10.1128/mBio.01970-16.1 FIG S1  CHIKV RNA synthesis is not affected by mutation of the capsid protein NoLS. BHK-21 and C6/36 cells were infected with CHIKV-WT or CHIKV-NoLS at an MOI of 0.1 PFU/cell. The viral genome copy number in the culture supernatants and viral RNA copy number in infected cells were determined by RT-qPCR at the indicated times postinfection. (A) BHK-21 cell-associated virus. (B) C6/36 cell-associated virus. (C) BHK-21 culture supernatant. (D) C6/36 culture supernatant. ***, *P* < 0.001, by two-way ANOVA with Bonferroni posttests. Each bar represents the mean ± standard error from 3 independent experiments. Download FIG S1, TIF file, 0.4 MB. Copyright © 2017 Taylor et al. 2017 Taylor et al. This content is distributed under the terms of the Creative Commons Attribution 4.0 International license.

### CHIKV-NoLS-infected mice show no signs of acute CHIKV disease.

**FIG 7:**
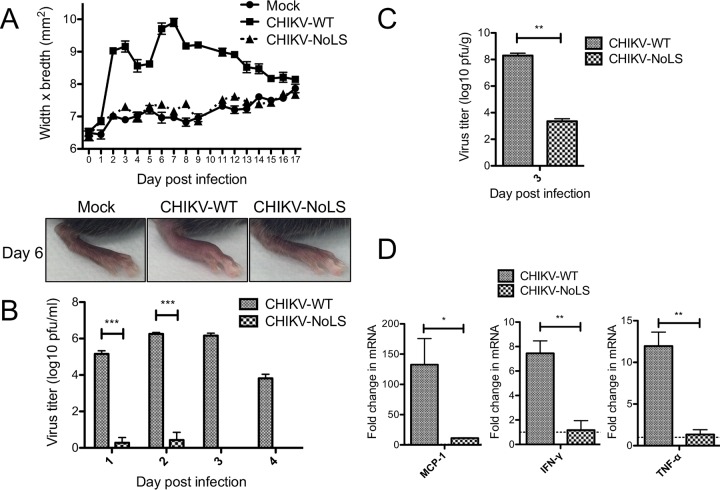
CHIKV-NoLS-infected mice show no signs of acute CHIKV disease. Twenty-one-day-old C57BL/6 mice were infected subcutaneously in the ventral/lateral side of the hind foot with 10^4^ PFU of CHIKV-WT or CHIKV-NoLS or mock infected with PBS alone. (A) CHIKV-induced footpad swelling was assessed daily by measuring the height and width of the perimetatarsal area of the hind foot. Each symbol represents the mean ± standard error from 5 to 6 mice. (B) At days 1, 2, 3, and 4 postinfection, serum was harvested and viremia determined. (C) Ankle tissues were harvested at day 3 postinfection and homogenized to determine amount of infectious virus by plaque assay on Vero cells. Each bar represents the mean ± standard error from 5 to 6 mice. ***, *P* < 0.001, by two-way ANOVA with Bonferroni posttests. (D) Expression of proinflammatory factors of acute CHIKV disease in ankle tissue was analyzed using RNA extraction and RT-qPCR. Results were normalized to the level of the housekeeping gene *HPRT1* and expressed as fold changes compared to the levels in the mock control samples (horizontal dotted line). *, *P* < 0.05, and **, *P* < 0.01, by Student’s unpaired *t* tests.

Studies examining the role of eastern equine encephalitis virus (EEEV) capsid protein in the inhibition of host gene expression during infection identified, through deletion analysis, amino acids in the N-terminal part of capsid protein important for virulence *in vivo* (18). To evaluate the virulence of a CHIKV unable to traffic capsid protein to the nucleolus, C57BL/6 mice were infected subcutaneously in the ventral/lateral side of the foot with 10^4^ PFU CHIKV-WT or CHIKV-NoLS or mock infected with phosphate-buffered saline (PBS) alone and monitored daily for signs of CHIKV-induced footpad swelling. Two peaks of footpad swelling were observed in CHIKV-WT-infected mice, at approximately days 2 and 6 postinfection, and swelling was fully resolved by day 17 postinfection (Fig. 7A). CHIKV-NoLS infected mice developed no footpad swelling (Fig. 7A). Viremia in CHIKV-NoLS-infected mice was significantly reduced at days 1 and 2 postinfection, compared to that in CHIKV-WT-infected mice and was below the limit of detection at days 3 and 4 postinfection (Fig. 7B). The amount of infectious virus recovered from the ankle tissue, close to the site of inoculation, of CHIKV-NoLS-infected mice was also significantly reduced compared to that in CHIKV-WT-infected mice at day 3 postinfection (Fig. 7C). Also, the activation of expression of key inflammatory mediators of CHIKV disease, monocyte chemoattractant protein-1 (MCP-1), interferon gamma (IFN-γ), and tumor necrosis factor alpha (TNF-α), was significantly less prominent in the ankle tissue of CHIKV-NoLS-infected mice compared to CHIKV-WT-infected mice at day 3 postinfection (Fig. 7D). Together, these results demonstrate that CHIKV-NoLS is highly attenuated in mice and that mutations in the NoLS of capsid protein diminish CHIKV virulence *in vivo*.

### CHIKV-NoLS-immunized mice are protected from CHIKV disease when challenged with CHIKV-WT.

**FIG 8:**
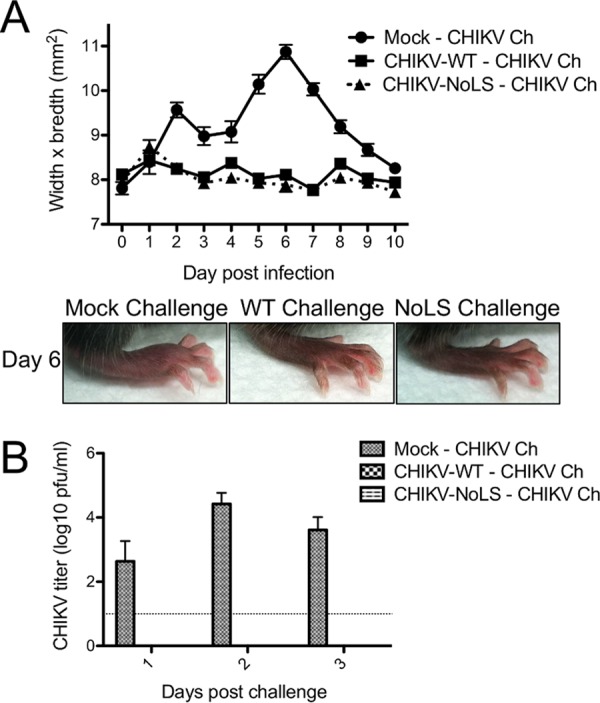
Mice immunized with CHIKV-NoLS are protected from disease when challenged with CHIKV-WT 30 days postimmunization. Twenty-one-day-old C57BL/6 mice were immunized subcutaneously in the ventral/lateral side of the hind foot with 10^4^ PFU of CHIKV-NoLS, infected with the same dose of CHIKV-WT, or mock infected with PBS alone. At 30 days postinfection, mice were challenged (Ch) in the ventral/lateral side of the hind foot with 10^4^ PFU of CHIKV-WT. Acute CHIKV disease upon challenge was assessed by measuring the height and width of the perimetatarsal area of the hind foot every 24 h (A). Each symbol represents the mean ± standard error from 5 to 6 mice. (B) Viremia was measured at days 1, 2, and 3 postchallenge. Each bar represents the mean ± standard error from 5 to 6 mice, and the horizontal dotted line represents the limit of detection.

As mutation of the NoLS of capsid protein dramatically reduced CHIKV virulence in infected mice, the highly attenuated CHIKV-NoLS may have potential as a CHIKV vaccine candidate. To examine the prophylactic efficacy of CHIKV-NoLS, mice were subcutaneously immunized in the ventral/lateral side of the foot with one dose of 10^4^ PFU CHIKV-NoLS; control mice were infected with the same dose of CHIKV-WT or mock immunized with PBS alone. At 30 days postimmunization, mice were challenged with 10^4^ PFU CHIKV-WT in the ventral/lateral side of the foot and monitored daily for signs of CHIKV-induced footpad swelling. Two peaks of footpad swelling, at days 2 and 6 postinfection, were observed in mock-immunized mice challenged with CHIKV-WT (Fig. 8A). Similar to CHIKV-WT-infected control mice, mice immunized with CHIKV-NoLS showed no signs of footpad swelling upon challenge with CHIKV-WT and developed no detectable viremia from days 1 to 3 postchallenge (Fig. 8B). Thus, CHIKV-NoLS immunization efficiently protected CHIKV-WT-challenged mice from the development of CHIKV-induced disease signs and detectable viremia.

As protection from alphaviral infection has been reported to be largely antibody driven, the neutralizing capacity of CHIKV-NoLS-induced antibodies was examined. Sera from mice immunized with 10^4^ PFU CHIKV-NoLS, infected with the same dose of CHIKV-WT or mock immunized with PBS alone, were collected at day 30 postimmunization. The neutralizing capacity of sera against CHIKV was rather similar in sera collected from mice immunized with CHIKV-NoLS or infected with CHIKV-WT at day 30 postimmunization (see Fig. S2 in the supplemental material). The highest concentration of serum (10^−1^) from both of these groups of mice preincubated with CHIKV effectively neutralized infectivity* in vitro*. The effect of sera from CHIKV-NoLS immunized mice was, however, somewhat less prominent than that of sera from CHIKV-WT-infected mice, most probably due to less effective spread (and correspondingly reduced amounts of antigen-producing cells) of the mutant virus. Nevertheless, antibodies induced by CHIKV-NoLS immunization were shown to efficiently neutralize CHIKV.

10.1128/mBio.01970-16.2 FIG S2  Neutralizing capacity of pooled sera from CHIKV-WT- or CHIKV-NoLS-immunized mice. Groups of 5 to 6 21-day-old C57BL/6 mice were immunized subcutaneously in the ventral/lateral side of the hind foot with 10^4^ PFU of CHIKV-NoLS, infected with the same dose of CHIKV-WT, or mock infected with PBS alone. Sera were collected at day 30 postimmunization, pooled, and heat inactivated. CHIKV-ZsGreen taken in an amount required for infection with an MOI of 0.4 was mixed with diluted (10^−1^, 10^−2^, and 10^−3^) serum. Virus-antibody mixtures were added to Vero cells, followed by incubation for 6 h. Infectivity was measured as percentage of ZsGreen-positive live cells. The horizontal dotted line represents the no-antibody control. ****, *P* < 0.0001, by one-way ANOVA with Bonferroni’s posttest comparison against the mock-infected control. Download FIG S2, TIF file, 0.1 MB. Copyright © 2017 Taylor et al. 2017 Taylor et al. This content is distributed under the terms of the Creative Commons Attribution 4.0 International license.

### CHIKV-NoLS immunization reduces early and peak viremia in RRV-challenged mice.

**FIG 9:**
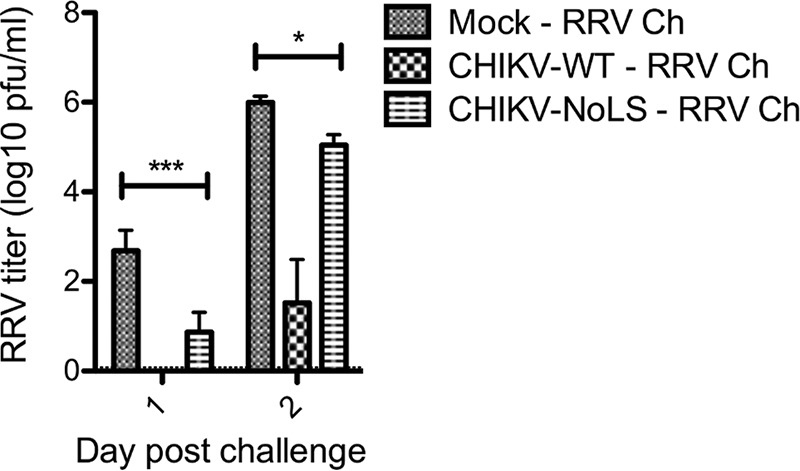
Mice immunized with CHIKV-NoLS show reduced viremia when challenged with RRV. Twenty-one-day-old C57BL/6 mice were immunized subcutaneously in the ventral/lateral side of the hind foot with 10^4^ PFU of CHIKV-NoLS, infected with the same dose of CHIKV-WT, or mock infected with PBS alone. At 30 days postinfection, mice were challenged (Ch) subcutaneously in the thorax with 10^4^ PFU of RRV. Viremia was measured at days 1 and 2 postchallenge, and the amount of infectious virus in challenged mouse sera was quantified by plaque assay. Each bar represents the mean ± standard error from 5 to 6 mice, and the horizontal dotted line represents the limit of detection. *, *P* < 0.05, and ***, *P* < 0.001, by two-way ANOVA with Bonferroni posttests.

A number of studies have highlighted the cross-protective effects of broadly neutralizing antialphavirus antibodies. To examine the cross-protective effect of CHIKV-NoLS against medically important alphaviruses other than CHIKV, mice immunized with 10^4^ PFU of CHIKV-NoLS, infected with the same dose of CHIKV-WT or mock immunized with PBS alone, were challenged with 10^4^ PFU of related arthritogenic alphavirus Ross River virus (RRV) subcutaneously in the thorax, and viremia was measured. CHIKV-NoLS-immunized mice show significantly reduced peak and early viremia, days 1 and 2 postchallenge, upon challenge with RRV (Fig. 9). Coherent with data from the neutralization assay, the protective effect of CHIKV-NoLS immunization was less prominent than that observed in CHIKV-WT-infected mice. Nevertheless, by reducing viremia, an indicator of disease outcome, CHIKV-NoLS has the potential to offer cross-protection against disease caused by other arthritogenic alphaviruses such as RRV.

## DISCUSSION

As positive-strand RNA viruses, alphaviruses replicate within the cytoplasm of host cells. Despite this, several alphaviruses target a number of viral proteins to the nucleus. The rationale for this is not immediately obvious; however, previous studies have highlighted host cell shutoff as one facet of capsid protein nuclear localization in encephalitic alphaviruses (20). Compartmentalization of viral proteins within subcellular structures of the host cell is therefore a mechanism used by alphaviruses to augment protein function and optimize infectivity, particularly for a protein with multiple roles during infection such as CHIKV capsid protein.

The nucleolus is a highly dynamic subnuclear structure. Typically thought of as the site of ribosome biogenesis, the nucleolus participates in numerous cellular processes, including transcriptional regulation, cell signaling, and response to stress (21, 22). Viruses from a variety of different families are now known to interact with the nucleolus during infection. Although the functional significance of viral nucleolar tropism is generally not well understood, a growing body of evidence suggests that viruses sequester cellular nucleolar proteins or target viral proteins to the nucleolus to facilitate viral replication (23–25). Mutations in the NoLS of porcine reproductive and respiratory syndrome virus N protein and Japanese encephalitis virus core protein have been shown to attenuate virus replication and reduce pathogenesis *in vivo*, making these mutants ideal live attenuated vaccine candidates (26, 27).

Deletion analysis has identified nuclear import and export signals within CHIKV capsid protein, and highlights the importance of the N-terminal part of the protein in nuclear import (10). Furthermore, deletion studies of the N-terminal part of eastern equine encephalitis virus (EEEV) capsid protein show that this region is critical for EEEV pathogenesis (18). The intracellular localization of capsid protein has been linked to encephalitic alphavirus attenuation previously in both *in vitro* and *in vivo* settings (15, 28). Here, we confirm nucleolar localization of CHIKV capsid protein using recombinant EGFP-tagged proteins. Using site-directed mutagenesis, we identify amino acid residues within the N-terminal part of CHIKV capsid protein responsible for nucleolar localization. Furthermore, disruption of the NoLS of CHIKV capsid protein was also found to dramatically inhibit capsid protein nuclear trafficking. It is likely that there is functional overlap between the trafficking motifs and NoLS located within the N-terminal portion of capsid protein. A substantial number of amino acid residues that make up the NoLS characterized herein were deleted in the previously defined nuclear localization signal of CHIKV capsid protein (10). Furthermore, the decreased nuclear trafficking ability of EGFP-Capsid-NoLS compared to EGFP-Capsid-101 seen at later time points (Fig. 3B) may be an accumulative effect from the additional mutations present in Capsid-NoLS.

Having identified the NoLS of capsid protein, we examined the importance of the region involved in capsid protein nucleolar localization for CHIKV replication. Immunofluorescence analysis established that in CHIKV-WT-infected Vero cells, capsid protein was accumulating in a subnuclear body, reminiscent of the nucleolus. In CHIKV-NoLS-infected Vero cells, such localization was not observed, suggesting the NoLS mutation was maintained in the infectious virus genome. However, in CHIKV-WT-infected mosquito (C6/36) cells, capsid protein subcellular localization was largely cytoplasmic, with no subnuclear puncta observed. Localization of CHIKV capsid protein to subnuclear domains is therefore not a characteristic of infection in all cell types. The replication of CHIKV-NoLS in mammalian (BHK-21) and mosquito (C6/36) cells was attenuated, producing significantly lower titers than CHIKV-WT. The overall reduced replication of CHIKV-NoLS observed in both IFN-α/β-deficient BHK-21 cells and mosquito cells suggests that mutation of the NoLS results in a defect in infectious virion formation and not in virus RNA production. This is likely linked to disruption of another essential function of capsid protein—for example, RNA binding and/or homomultimerization. We have shown that nucleolar localization is not required for the autoproteolytic cleavage of capsid protein; however, the N-terminal region of capsid protein is also thought to be important for nucleocapsid formation (8). Further molecular studies are required to determine whether the region responsible for nucleolar localization of CHIKV capsid protein, or even nucleolar localization of the protein itself, is required for RNA binding and/or homomultimerization. Viral protein nucleolar localization is thought to be intimately linked to viral protein-RNA and protein-protein interactions in a number of viruses from various families. Attenuated replication in insect cells is, however, an important safety feature for any live attenuated arbovirus vaccine candidate, to avoid infection of viral vectors.

CHIKV-NoLS-infected mice show no signs of acute CHIKV disease, highlighting the importance of the region responsible for capsid protein subcellular localization to the replication and pathogenicity of an arthritogenic alphavirus. The dramatic effect of the NoLS mutation in capsid protein on CHIKV virulence in mice led us to investigate the suitability of CHIKV-NoLS as a live attenuated vaccine candidate. Mice immunized with CHIKV-NoLS showed no signs of footpad swelling upon challenge with CHIKV-WT at day 30 postimmunization and developed no detectable viremia from days 1 to 3 postchallenge. Immunization with CHIKV-NoLS protected mice from CHIKV challenge for up to 30 days, and antibodies induced by CHIKV-NoLS immunization efficiently neutralize CHIKV *in vitro*, indicating CHIKV-NoLS is immunogenic after one dose. Further long-term studies will assess whether CHIKV-NoLS-induced immunity is long-lived. A growing body of evidence, including our own studies, indicates cross-reactivity of alphavirus antibodies with broadly neutralizing effects both *in vitro* and *in vivo* (29–32). Cross-protection against a number of other arthritogenic alphaviruses is a desirable feature of a CHIKV vaccine candidate. CHIKV-NoLS-immunized mice show significantly reduced peak and early viremia upon challenge with related alphavirus RRV. By reducing viremia, an indicator of disease outcome, CHIKV-NoLS has the potential to offer cross-protection against disease caused by other arthritogenic alphaviruses.

The identification of mutations able to specifically inhibit capsid protein nucleolar localization is of great importance in evaluating the roles of nucleolar interactions during alphaviral infection, as well as demonstrating the potential of these interactions as targets for the development of novel antiviral therapies that target viral protein subcellular trafficking or aid the design of novel live attenuated vaccine candidates against CHIKV and other arthritogenic alphaviruses. Additional studies will be required to investigate the safety profile, level of attenuation, and long-term vaccine efficacy of CHIKV-NoLS or similar mutant viruses to allow further development of a live attenuated vaccine candidate.

## MATERIALS AND METHODS

### Oligonucleotides, plasmids, and antibodies.

To generate pCapsid-EGFP, cDNA corresponding to CHIKV capsid protein was amplified by PCR using primers CHIKCprotF (5′ GCGGCGCAAGCTTATGGAGTTCATCCCAACCC 3′) and CHIKCprotR (5′ CGCGGATCCGACTCTTCGGCCCCCTCG 3′) and cloned into pEGFP-N1 (TaKaRa Bio, Inc., USA). To generate pCapsidW-EGFP containing the tryptophan residue required for capsid protein autoproteolytic cleavage at the C-terminal part of capsid protein, primers CHIKCprotF and CHIKCprotWR (5′ CGCGGATCCGACCACTCTTCGGCC 3′) were used, and the obtained fragment was cloned into pEGFP-N1. pSP6-CHIKV-ZsGreen, a plasmid containing cDNA of a CHIKV variant expressing the ZsGreen marker protein, was constructed using a full-length infectious cDNA clone of the La Reunion CHIKV isolate LR2006-OPY1 as described previously (33). The oligonucleotides used in site-directed mutagenesis are listed in Table S1 in the supplemental material. Mutants were generated using a QuikChange II site-directed mutagenesis kit (Agilent Technologies, Inc., USA). Antibodies to nucleolin (Santa Cruz Biotech, Inc., USA), EGFP (BD Biosciences, USA), and actin (Santa Cruz Biotech, Inc., USA) were purchased from the respective suppliers. Monoclonal capsid protein antibody was made in-house and characterized as described previously (34, 35). A cocktail of anticapsid monoclonal antibodies (1.7B2 and 4.1H11) was used for immunofluorescence.

10.1128/mBio.01970-16.3 TABLE S1  Primers used in site-directed mutagenesis of CHIKV capsid protein. Download TABLE S1, DOCX file, 0.1 MB. Copyright © 2017 Taylor et al. 2017 Taylor et al. This content is distributed under the terms of the Creative Commons Attribution 4.0 International license.

### Cell culture, transfection, and virus propagation.

Vero and BHK-21 cells were cultured in Opti-MEM (Gibco, Thermo Fisher Scientific, Australia), supplemented with 3% fetal calf serum (FCS). C6/36 cells were cultured in Leibovitz's L-15 medium (Gibco, Thermo Fisher Scientific, Australia), supplemented with 10% tryptose phosphate broth and 10% FCS. Plasmid transfections were carried out with Lipofectamine 2000 (Thermo Fisher Scientific, Australia) according to the manufacturer’s instructions.

### Mice.

C57BL/6 WT mice were obtained from the Animal Resources Centre (Perth, Australia) and bred in-house. All animal experiments were performed in accordance with the guidelines set out by The Griffith University Animal Ethics Committee. Twenty-one-day-old C57BL/6 male and female mice, in equal distribution, were inoculated in the ventral/lateral side of the foot with 10^4^ PFU CHIKV-WT or CHIKV-NoLS diluted in PBS to a volume of 20 µl. Mock-infected mice were inoculated with PBS alone. Mice were weighed and scored for disease signs every 24 h and sacrificed by CO_2_ asphyxiation at experimental endpoints. CHIKV-induced footpad swelling was assessed by measuring the height and width of the perimetatarsal area of the hind foot, using Kincrome digital vernier calipers. At 30 days postinfection, mice were challenged in the ventral/lateral side of the foot with 10^4^ PFU CHIKV-WT, weighed, and scored for disease signs every 24 h, with viremia measured at days 1, 2, and 3 postchallenge, or 10^4^ PFU Ross River virus (RRV) subcutaneously in the thorax, with viremia measured at days 1 and 2 postchallenge.

### Neutralization assay.

The neutralizing capacity of antibody from CHIKV-WT- or CHIKV-NoLS-infected mice at day 30 postinfection was analyzed by immunofluorescence-based cell infection assays using Vero cells and CHIKV-ZsGreen. Infectious virus, taken at an amount sufficient for a multiplicity of infection (MOI) of 0.4, was mixed with diluted (10^−1^, 10^−2^, and 10^−3^), heat-inactivated (56°C for 30 min) pooled mouse serum, followed by incubation for 2 h at 37°C. Virus-antibody mixtures were added to Vero cells and incubated at 37°C for 1 h. The virus inoculum was removed, cells were washed with PBS, and Opti-MEM containing 3% FCS was added, followed by incubation for 6 h at 37°C. Cells were gently resuspended, stained with LIVE/DEAD near-infrared cell stain (Thermo Fisher Scientific, Australia), and fixed in 4% paraformaldehyde. Infectivity was measured as percentage of ZsGreen-positive live cells using a BD LSR II Fortessa cell analyzer and quantified with FlowJo software (Treestar, Inc., USA).

### Immunofluorescence microscopy and FLIP.

Cells grown on polylysine-treated coverslips were fixed in 4% paraformaldehyde and permeabilized in 1% Triton X-100. Cells were then blocked in 1% bovine serum albumin (BSA) made in PBS and incubated at 37°C for 1 h. Primary antibodies were diluted 1:100 in 1% BSA and incubated with the cells for 1 h at 37°C. Alexa Fluor 647-conjugated secondary antibody (Invitrogen, Thermo Fisher Scientific, Australia), was diluted 1:500 in 1% BSA and incubated with the cells for 1 h at 37°C. Coverslips were mounted in Vectorshield mounting medium (Vector Laboratories, USA), and staining was visualized on an Olympus FluoView FV1000 confocal microscope. For FLIP analysis, Vero cells were plated on glass-based 33-mm culture dishes and imaged at 24 h posttransfection using an LSM 510 Meta confocal microscope (Zeiss, Oberkochen, Germany). Cells were maintained at 37°C, and during imaging, the cell culture medium was exchanged for CO_2_-independent medium (Invitrogen, Thermo Fisher Scientific, Australia). Fluorescence loss at the region of interest (ROI) was normalized using the relative fluorescence intensity from the LSM 510 software; initial fluorescence intensity was set as 1.

### *In vitro* viral replication kinetics.

BHK-21 and C6/36 cells were infected with CHIKV-WT or CHIKV-NoLS at an MOI of 0.1 and allowed to incubate for 1 h at 37°C in a 5% CO_2_ incubator before virus was removed and the cells were washed with PBS and overlaid with Opti-MEM containing 3% FCS. At various times postinfection, supernatant aliquots were harvested and viral titers measured by plaque assay as outlined below. To determine the virus RNA genome copy number in culture supernatants and virus positive-strand RNA copy number in infected cells, supernatant was collected and monolayers washed three times in PBS. RNA extraction was performed using TRIzol (Invitrogen, Thermo Fisher Scientific, Australia), according to the manufacturer’s instructions. Extracted RNA was reverse transcribed using random nonamer primers and Moloney murine leukemia virus (MMLV) reverse transcriptase (Sigma-Aldrich, Inc., USA) according to the manufacturer’s instructions. The standard curve was generated using serial dilutions of a full-length infectious cDNA clone of the La Reunion CHIKV isolate LR2006-OPY1. Quantification of viral load was performed using SYBR Green real-time PCR reagent in 12.5-μl reaction volume to detect the E1 region. Primers CHIKV E1F (5′ CCCGGTAAGAGCGGTGAA 3′) and CHIKV E1R (5′ CTTCCGGTATGTCGATG 3′) were used to detect CHIKV genomic, antigenomic, and subgenomic RNAs. All reactions were performed using a CFX96 Touch real-time PCR system. The standard curve was plotted, and copy numbers of amplified products were interpolated from a standard curve using GraphPad Prism software to determine viral RNA copy number.

### Viral titer assay.

Mice were sacrificed at days 1, 2, 3, and 4 postinfection, with the ankle joint and serum collected and assayed for viral titer using a plaque assay. Tissue samples were homogenized in 1 ml of PBS, and 10-fold serial dilutions of homogenate and sera were added in triplicate to Vero cells. Virus was allowed to incubate for 1 h at 37°C in a 5% CO_2_ incubator before the virus was removed and the cells overlaid with Opti-MEM containing 3% FCS and 1% agarose (Sigma-Aldrich, Inc., USA) and incubated for 48 h in a 5% CO_2_ incubator. Cells were fixed in 1% formalin, and virus plaques were made visible by staining with 0.1% crystal violet. Results are expressed as PFU per milliliter or PFU per gram of tissue.

### RT-qPCR.

RNA was extracted from tissues using TRIzol (Invitrogen, Thermo Fisher Scientific, Australia), according to the manufacturer’s instructions. One microgram of total RNA was reverse transcribed using random nonamer primers and MMLV reverse transcriptase (Sigma-Aldrich, Inc., USA) according to the manufacturer’s instructions. Reverse transcription-quantitative PCR (RT-qPCR) was performed with 50 ng of template cDNA, QuantiTect primer assay kits (Qiagen, Hilden, Germany), and SYBR Green real-time PCR reagent in a CFX96 Touch real-time PCR system using a standard three-step melt program (95°C for 15 s, 55°C for 30 s, and 72°C for 30 s). Data were normalized to the *HPRT1* housekeeping gene, and the fold change in mRNA expression relative to mock-infected PBS-treated samples for each gene was calculated using the threshold cycle (ΔΔ*C*_*T*_) method: ΔΔ*C*_*T*_ = Δ*C*_*T*_ of virus infected − Δ*C*_*T*_ of mock infected, where Δ*C*_*T*_ = *C*_*T*_ of gene of interest − *C*_*T*_ of housekeeping gene. The fold change for each gene is calculated as 2^−ΔΔ*CT*^.

### Statistical analysis.

Two-way ANOVA with Bonferroni posttests was used to examine *in vitro* viral growth kinetic data and viremia. Student’s unpaired *t* tests were used to analyze RT-qPCR and ankle titers at day 3 postinfection. One-way ANOVA with Bonferroni posttests was used to examine the neutralization assay. A *P* value of <0.05 was considered to be significant.
