# Supplementary figures and images for: Mutation of the N-Terminal Region of Chikungunya Virus Capsid Protein: Implications for Vaccine Design
Source: mBio. 2017 Feb 21;8(1):e01970-16. doi: 10.1128/mBio.01970-16 (PMC5358915; doi:10.1128/mBio.01970-16)

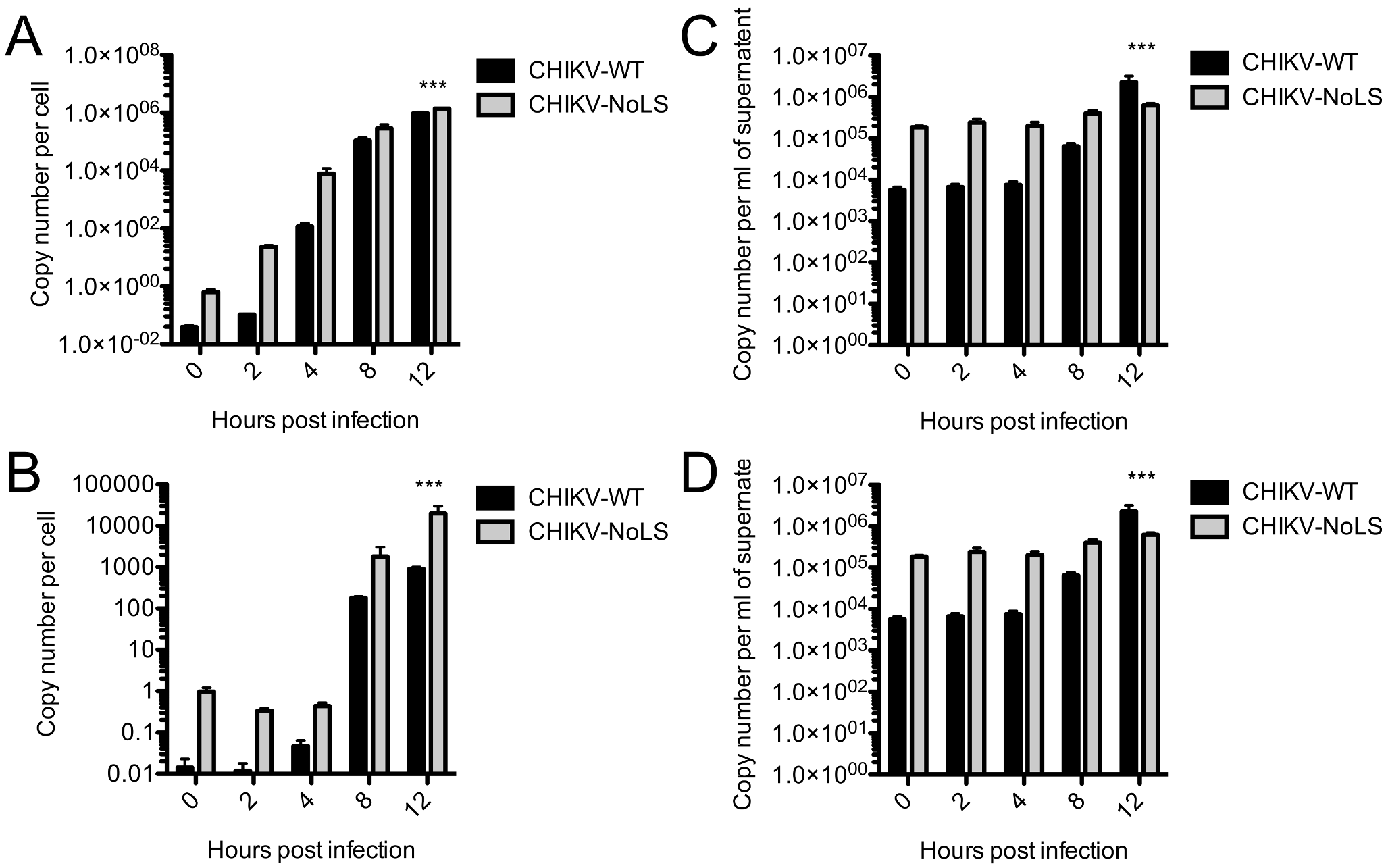

Supplement: FIG S1 [file mbo002173152sf1.tif]

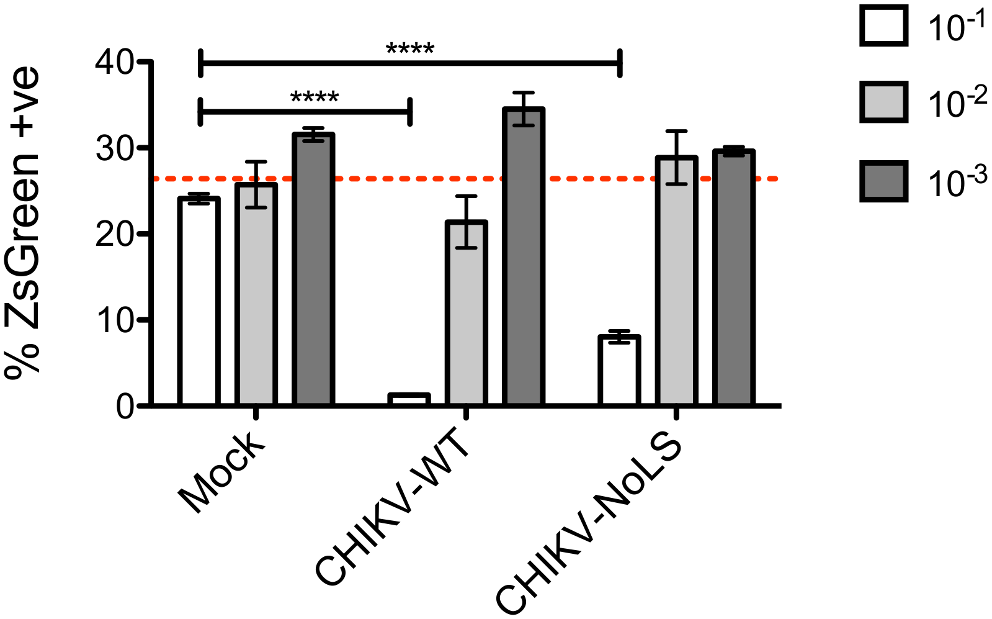

Supplement: FIG S2 [file mbo002173152sf2.tif]
