# Supplementary material for: Mutation of the N-Terminal Region of Chikungunya Virus Capsid Protein: Implications for Vaccine Design
Source: mBio. 2017 Feb 21;8(1):e01970-16. doi: 10.1128/mBio.01970-16 (PMC5358915; doi:10.1128/mBio.01970-16)
Supplement: TABLE S1 [file mbo002173152st1.docx]

| **Primer name** | **Sequence** |
| --- | --- |
| K84/85A sense | 5′ caaaacaacacaaatcaagcggcgcagccacctaaaaagaaac |
| K84/85A antisense | 3′ gttttgttgtgtttagttcgccgcgtcggtggatttttctttg |
| K95/96A sense | 5′ gaaaccggctcaagcggcaaagaagccgggc |
| K95/96A antisense | 3′ ctttggccgagttcgccgtttcttcggcccg |
| R101/102A sense | 5′ gaagccgggcgccgcagagaggatgtgcatgaaaatcg |
| R101/102A antisense | 3′ cttcggcccgcggcgtctctcctacacgtacttttagc |
| R62/63A sense | 5′ gcggtaccccaacagaagccagccgcgaatcggaagaataag |
| R62/63A antisense | 3′ cgccatggggttgtcttcggtcggcgcttagccttcttattc |
| K68/69A sense | 5′ gaatcggaagaatgcggcgcaaaagcaaaaacaacaggcgcc |
| K68/69A antisense | 3′ cttagccttcttacgccgcgttttcgtttttgttgtccgcgg |
| RK65/66A sense | 5′ cagccgcgaatgcggcgaatgcggcgcaaaag |
| RK65/66A antisense | 3′ gtcggcgcttacgccgcttacgccgcgttttc |
